# Supplementary material for: Effects of multitask training on cognition and motor control in people with schizophrenia spectrum disorders
Source: PLoS One. 2022 Jun 30;17(6):e0264745. doi: 10.1371/journal.pone.0264745 (PMC9246115; doi:10.1371/journal.pone.0264745)
Supplement: S1 File — (PDF) [file pone.0264745.s001.pdf]

ClinicalTrials.gov PRS DRAFT Receipt (Working Version)
Last Update: 11/17/2020 20:06

ClinicalTrials.gov ID: [Not yet assigned]

Study Identification

Unique Protocol ID: NCT04629898
ERROR : "NCT04629898" does not refer to this record.

Brief Title: Level of Immersion of Virtual Reality and Cognition and Motor Performance in Patients of Schizophrenia Spectrum Disorder

Official Title: The Influence of Level of Immersion on Training Effects of Exergaming for Persons With Schizophrenia Spectrum Disorder

Secondary IDs:

Study Status

Record Verification: November 2020

Overall Status: Completed

Study Start: August 1, 2017 [Actual]

Primary Completion: July 31, 2018 [Actual]

Study Completion: July 31, 2018 [Actual]

Sponsor/Collaborators

Sponsor: National Taiwan Normal University

Responsible Party: Principal Investigator
Investigator: Jen-Suh Chern [jschern]
Official Title: Associate Professor
Affiliation: National Taiwan Normal University

Collaborators:

Oversight

U.S. FDA-regulated Drug: No

U.S. FDA-regulated Device: No

U.S. FDA IND/IDE: No

Human Subjects Review: Board Status: Approved
Approval Number: 201612HM014
Board Name: Center for Research Ethics
Board Affiliation: National Taiwan Normal University
Phone: +886-2-77491395
Email: kinkilover@ntnu.edu.tw
Address:

162, Sec. 1, Heping E. Rd., Da-An District, 10622, Taipei City, Taiwan

Data Monitoring: No

FDA Regulated Intervention: No

Study Description

Brief Summary: Schizophrenia Spectrum Disorder (SSD) is the main diagnosis in subjects with mental disability. The number of disabled SSD increased yearly and might be due to the ineffective medical and rehabilitative interventions on improving cognitive and motor function. Innovative and effective training programs are needed to decrease financial burden of medical care and social welfare.

Multi-tasks in real world were found to be effective in improving the sports skill of athletes and multi-tasking ability in the real world. No any study investigates in depth how the multi-tasks affect SSD patients' cognitive and motor performance.

Virtual reality exergaming (VREG) is a new form of multi-tasks incorporating information technology. It has been proved as an effective intervention media for patients with SSD in the single domain of physical fitness. No study exists examining its simultaneous influence in cognition and motor performance in SSD patients.

The purpose of this study is to examine the effects of different forms of multi-tasks on the cognitive and motor performance. The results of this study might be used by clinician treating patients with SSD in clinical decision making regarding to whether or not incorporating contemporary information technology in daily intervention and, furthermore, benefit the SSD more than using only the multi-task in the real world.

A total of 25 patients were recruited and participated in this study. They underwent 2-stage multi-task training. The first stage training used multi-tasks in the real world; the second stage training used new forms of multi-tasks which is virtual reality video sport games conveyed by Xbox Kinect. The training in each stage last for 12 weeks and there were 2 sessions in each week. The training duration in each session was 40 minutes.

All participants were measured at the following three time points: before any training begins, after the 1st stage of training and after the 2nd stage of training. The outcome measures include: upper limb motor function (measured by Box-and-Block Test), cognitive function (measured by Color Trail Test), functional mobility measured by Timed-Up-and-Go Test, and standing stability in 6 stance conditions, and voluntary center of gravity shifts during Functional Reach Test (measured by Footscan pressure measurement system).

Detailed Description: NOTE : Detailed Description has not been entered.

Conditions

Conditions: Schizophrenia
Virtual Reality
Video Games
Multi-tasks

Keywords:

Study Design

Study Type: Interventional

Primary Purpose: Health Services Research

Study Phase: N/A

Interventional Study Model: Single Group Assignment

Number of Arms: 1

Masking: Single (Investigator)

Allocation: Non-Randomized

Enrollment: 25 [Actual]

Arms and Interventions

Intervention Details:

Behavioral: multi-task in the real world and virtual reality video sport games

This was a 2-staged intervention study. The 1st stage intervention uses multitasks in the real world including stepping exercise, ball-kicking activity and bean-bag tossing activity. The 2nd stage intervention used video sport games conveyed by Xbox Kinect. Tennis and golf games were selected.

⚠ WARNING : No Arms have been specified for this study.

Outcome Measures

Primary Outcome Measure:

1. The changes of Color Trail Test (CTT)
   There are two subtests: CTT1 & CTT2. The time spend to complete the two subtest is used representing executive function of the participants.

   [Time Frame: Measured at one week before and after the 1st stage training begin and one week after the end of the 2nd stage intervention]
2. The changes of Box-and-Block Test
   This is a measure of upper limb motor performance. The number of blocks moved in one minute is the score of performance.Both the right and the left arm are tested.

   [Time Frame: Measured at one week before and after the 1st stage training begin and one week after the end of the 2nd stage intervention]
3. The changes of Timed-Up-and-Go Test
   The time spend on completing the following task: standing up from a armless chair, walking ahead for 3 meteters, walking around a stool, walking back to the original chair and sitting down.

   [Time Frame: Measured at one week before and after the 1st stage training begin and one week after the end of the 2nd stage intervention]
4. The changes of Postural sway while standing quietly
   The participants are instructed to stand on a 0.5 meter pressure measurement plate in 3 different standing posture (bilateral foot shoulder width apart, bilateral feet side by side, and tandem stance) and two visual conditions (eyes open v.s. closed. Coordinates of center of pressure(COP) are recorded and output and the the total path length of COP is calculated. The longer the COP path length, the poorer the postural stability.

   [Time Frame: Measured at one week before and after the 1st stage training begin and one week after the end of the 2nd stage intervention]
5. The changes of Voluntary postural sway

The participants are instructed to stand on a 0.5 meter pressure measurement plate when performing the Functional Reach Test. Coordinates of center of pressure(COP) are recorded and output and the total path length of COP is calculated. The longer the COP path length, the better the dynamic postural control.

[Time Frame: Measured at one week before and after the 1st stage training begin and one week after the end of the 2nd stage intervention .]

Eligibility

Minimum Age: 18 Years

Maximum Age:

Sex: All

Gender Based: No

Accepts Healthy Volunteers: No

Criteria: Inclusion Criteria:

- (1) diagnosed as schizophrenia or schizoaffective disorder by senior psychiatrists according to DSM-IV criteria and the disease onset was >1 year ago, (2) been judged as symptomatically stable by therapists, (3) was free of interfering or uncontrolled behaviors, (4) could stand independently for at least 3 minutes and walk 10 m without any mobility aids, (5) was ≥18 years old during recruitment for this study, (6) willing to participate in the study voluntarily

Exclusion Criteria:

- Patients were excluded if they were diagnosed with musculoskeletal impairment in the past year, had significant visual and auditory impairments, with severe cognitive impairment (based on Mini Mental State Examination score < 18), or were unable to follow verbal instructions or training and evaluation regimen.

Contacts/Locations

Central Contact Person: Jen-Suh Chern, PhD
Telephone: +886-2-77495016
Email: chern8616@gmail.com

Central Contact Backup:

Study Officials: Jen-Suh Chern, PhD
Study Principal Investigator
National Taiwan Normal University

Locations: **Taiwan**
National Taiwan Normal University
Taipei, Taiwan
Contact: Yu-Jung Tsai +886919196430 joyce7522@gmail.com
Contact: Ext. Tsai joyce7522@gmail.com

IPDSharing

Plan to Share IPD: No

References

Citations:

Links:

Available IPD/Information:
