# Supplementary material for: Effects of multitask training on cognition and motor control in people with schizophrenia spectrum disorders
Source: PLoS One. 2022 Jun 30;17(6):e0264745. doi: 10.1371/journal.pone.0264745 (PMC9246115; doi:10.1371/journal.pone.0264745)
Supplement: S2 File — (PDF) [file pone.0264745.s002.pdf]

CONSORT
TRANSPARENT REPORTING of TRIALS

CONSORT 2010 Flow Diagram

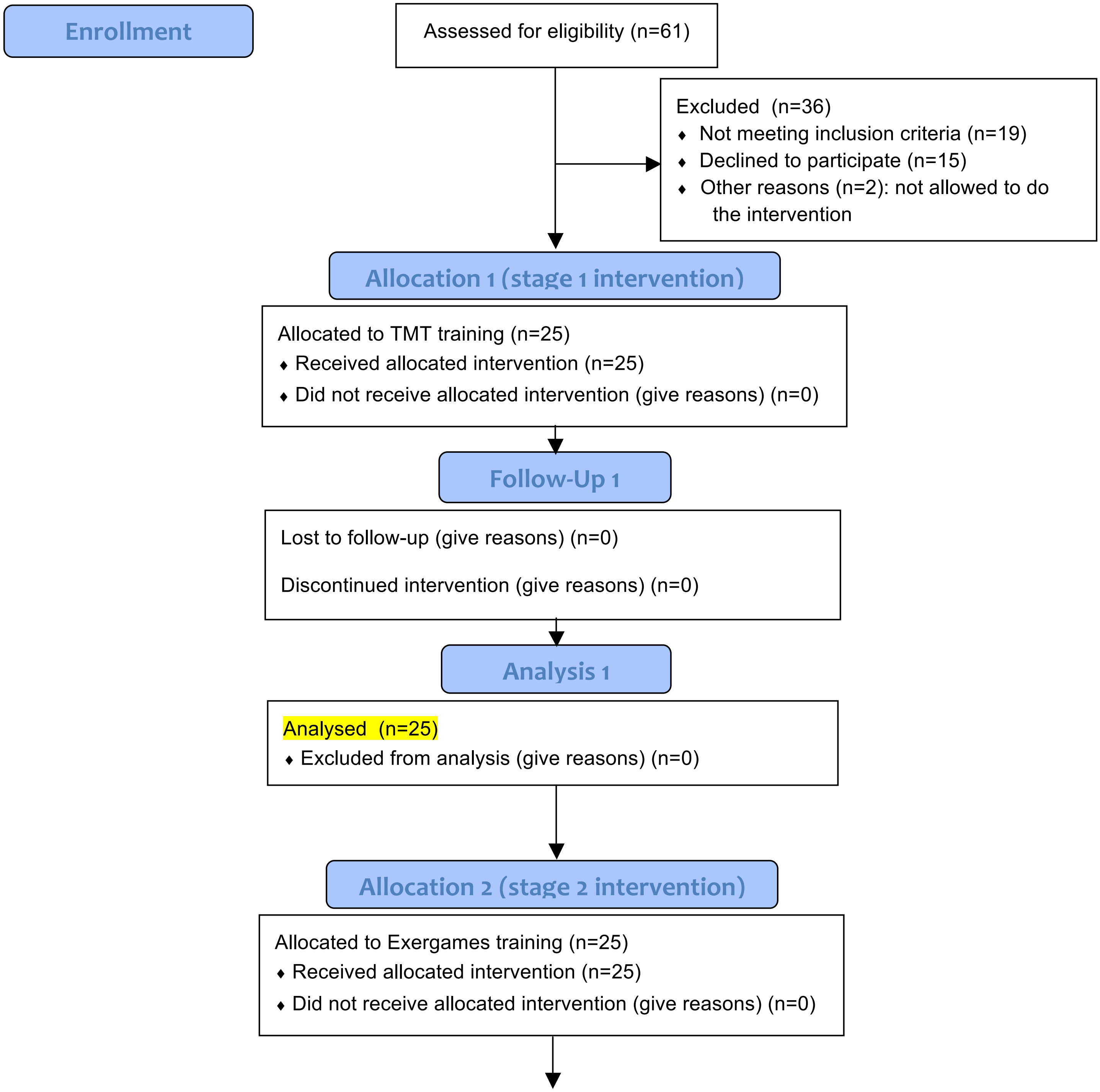

Follow-Up 2

Discontinued intervention (n=3)

- Psychiatric symptom unstable (n=1)
- Musculoskeletal diagnosis (n=1)
- No willingness o participate the research (n=1)

Analysis 2

Analysed (n=25)

♦ Excluded from analysis (give reasons) (n=0)
