# Supplementary material for: Effects of multitask training on cognition and motor control in people with schizophrenia spectrum disorders
Source: PLoS One. 2022 Jun 30;17(6):e0264745. doi: 10.1371/journal.pone.0264745 (PMC9246115; doi:10.1371/journal.pone.0264745)
Supplement: Attachment — Submitted filename: Response to Reviewers.pdf [file pone.0264745.s006.pdf]

Responses to Reviewers

Dear Academic Editor:

Thank you for your and reviewers' review and the resubmission opportunity. The following table shows the authors' point to point response to each reviewer's (Reviewer #1 and Reviewer #3) comments. Each comment is highly appreciated and the authors response to each comment with careful consideration.

Several typos are also corrected as shown in the file labeled "Revised Manuscript with Track Changes". Reference numbered 3 is added according to Reviewer#3's suggestions. References numbered 29 and 30 are used to replaced references numbered 28 and 29 according to Reviewer#3's suggestions

Thank you very much for your review and looking forward to hearing from you soon.

Best Wishes,

Jen-Suh Chern, PhD, Associate Professor
National Taiwan Normal University
Graduate Institute of Rehabilitation Counseling

Reviewers' Comments	Response from the authors
Reviewer #1:	
The authors corrected the article in accordance with the comments. Thank you very much for the opportunity to evaluate the article	Thank you for your review and making this manuscript better.
Reviewer #2:	
Table 3, a, b, c to be denoted in the table footnote. n for Pre, Po1, Po2 to be stated.	The a, b, c, in table 3 is denoted in the table footnotes. The n for Pre, Po1, Po2 is stated in table 3 is stated. (Line 475—482)
Line 430, adjustment with multiple comparisons, Bonferroni to be stated.	The Bonferroni is stated in line 425.
Figure 2. what is the small box beside re-training measurement?	The small box that the reviewer saw in figure might be format problem due to that the author is using the Apple Mac computer. The figure 2 has been check in a Microsoft computer and only essential information is presented.
Reviewer #3:	
Dear Authors,<br>Thank you for addressing my previous comments. The manuscript presents in much more accurate way the study. I have some additional comments to the revised version of the manuscript	Thank you for your review. The followings are the point-to-point response to your insightful comments.

Abstract:	
Line 54: Please re-phrase the results report on effectiveness of “both” interventions according to the study design. You actually can conclude about the first intervention effectiveness and about combination of the first and second together.	It has been revised as “ TMT and the combination of TMT and VGMT improved SSD patients’ cognition, ….”. (Line 54)
Introduction	
Line 69: “The neuropsychological system..” Please elaborate on connection between this sentence and the following one. Reference for this statement should be added.	The connection of the two sentences was made (Line 76-77) and the reference is added in line 76.
Lines 74-75: “Additionally….” Please check wording of the sentence. It sounds less logical.	“The neuropsychological system” is used to replace “agent” to make the sentence sounds logical. (Line 76)<br><br>“ In addition to maintaining the quality of life in the real world, the multitasking in ………of the neuropsychological system and motor apparatus” is revised as” the process is not only critical for multitask performance and maintaining quality of life in the real world, but also the multitasking…..of the neurological system and motor apparatus”. (Line 72—75)<br><br>The above two revision makes the sentences sound more logical.
Line 85: “balance” – please replace with balance	The “balanc” was a typo. It has been corrected into “balance”. (Line 87)
Line 109: double “and”. Please delete one of them.	The duplicate word “and” is deleted. (Line 112)
Lines 119 - 120 : “Impairments in cognitive functions usually occur during the first SSD episode” Please add more updated references.	More updated references are used to replace the original ones. (Line123)
Lines 124-127: “scores on the Minimal 125 Mental Status Evaluation (MMSE) … functions” should be removed to the discussion section.	The sentence has been removed. (Line 126)
Lines 137-141: “. Furthermore, Hidese and… dysfunction” is less relevant for the study.	The sentence has been deleted. (Line 137)

Please delete from the manuscript. You can provide direct references on brain functioning alteration in schizophrenia. It is well established factor.	
Lines 177-179: “Therefore, the purpose of this study was to examine the differential effects 178 between traditional goal-directed multitasking activities and video games on cognition and 179 upper extremity and postural control in people with SSD.” The goal should be rephrased since the study design not allow you to draw conclusion on differentiation between two types of the training.	The purpose of the study is rephrased as “ the purpose of this study was to examine the effects of traditional multitask training (TMT) and combining TMT and …….”. (Line 176—178)
Lines 179-182: You should rephrase the sentence according with the understanding of your study design.<br>Measurements	“ We predicted that the TMT and combining both forms of multitasking training with an increased training dosage ……” is used to replace the sentence in the original manuscript. (Line 179, 180)
Lines 318-319: Please remove the report on the blinding group allocation since there is no groups in the study.	Thank your for the comments. The sentence has been deleted. The original second paragraph is combined with the first paragraph. (Line 315—321))
Results	
Line 466: “indicated that VGMT further improved TUG scores significantly…” Please state clear that it is “VGMT training following TMT training”. There is no group with VGMT training only or first, so you have no data to relay on concluding about the VGMT training only.	It is rephrased as the followings: “the pairwise comparison indicated that VGMT training following TMT training …” (Line 461)
Line 469: “especially the form of video games” The same comment as previous. Please rephrase.	It is rephrased as: ”especially the form of video games following tradition multitasks ($p < 0.05$).” (Line 464)
Line 476: “multitasking in the form of video game” – The same as previous. You can speak about combination of two interventions. Please rephrase you reports throughout the manuscript.	It is rephrased as: “ pariwise comparison showed that multitasking in the form of videogame following traditional multitasks ….” and “compared with TMT” is removed. (Line 473)

	The whole manuscript is revised to show that the effects were not originated from VGMT only but from VGMt following TMT. For example line 564.
Discussion	
Lines 498 – 504: The same sentence duplicated twice. Please remove one of them.	The duplicated sentence is removed.(Line 503)
Line 509: “Therefore, ….” – please reword the sentence since it is not clear . “aht” - should be deleted.	“(traditional multitasking and video games)” in line 508 is revised as “ traditional multitasking followed by video games)” to avoid confusion.<br><br>The sentence “ the training effects might be due to the multitasks nature of the CTT. The CTT requires a participants …..”. to make it clearer. (Line 504, 505.)<br><br>The sentence “ Therefore, the authors believe that the tasks in this test are multitasks fundamentally” is revised as” therefore, the authors believe that the tasks in CTT are multitask in nature.” (Line 507, 508)
Lines 513-516: “The difficulty faced by people with SSD in multitasking was independent of the deficits in general processing speed, working memory, and attention.” You should support this statement with statistic data. Please add statistical support for your statement.	The sentence is deleted since it was confusion and there is no statistical data supporting it. (Line 511)
Lines 515-517: “Conversely, multitasking difficulties …” the same comments as the previous one.	The sentence is revised: “Multitasking difficulties in these population were related to complex cognitive functions across multiple domains, including cognitive flexibility, inhibitory function, planning ability, prospective memory and attention shifting as measured by CTT.” (Line 511—514)
Lines 536-537: “Hidese and colleagues [20] … impairment and decreased insular volume and white matter in people with SSD [17]. “ Please delete this sentence since it has little contribution to the topic of the manuscript.	The sentence is deleted. (Line 533)

Please add to the limitations that you have small sample size.	The small sample size is added as one of the limitations of this study. (Line 621)
