# Supplementary material for: Effects of multitask training on cognition and motor control in people with schizophrenia spectrum disorders
Source: PLoS One. 2022 Jun 30;17(6):e0264745. doi: 10.1371/journal.pone.0264745 (PMC9246115; doi:10.1371/journal.pone.0264745)
Supplement: Attachment — Submitted filename: Response to Reviewers.pdf [file pone.0264745.s007.pdf]

Responses to Reviewers

Dear Academic Editor:

Thank you for your and reviewers' review and the resubmission opportunity. The following table shows the authors' point to point response to each reviewer's (Reviewer #1 and Reviewer #3) comments. The line numbers in the table are numbers in the cleared Manuscript. Each comment is highly appreciated and the authors response to each comment with careful consideration.

Several typos are also corrected as shown in the file labeled "Revised Manuscript with Track Changes". Reference numbered 3 is added according to Reviewer#3's suggestions. New references numbered 29 and 30 are used to replaced references numbered 28 and 29 in the last version manuscript according to Reviewer#3's suggestions.

The corresponding author checked the title page again and decided to remove the funding statement from the title page to conform to the submission format of your journal. Please replace the funding statement on the online submission form with the following statement:

"This research was funded by Ministry of Science and Technology of Taiwan (https://www.most.gov.tw) grant number MOST-107-2410-H-003-126 and National Taiwan Normal University(https://www.ntnu.edu.tw) grant number106000131.The funders had no role in study design, data collection and analysis, decision to publish, or preparation of the manuscript."

Thank you very much for your review and looking forward to hearing from you soon.

Best Wishes,

Jen-Suh Chern, PhD, Associate Professor
National Taiwan Normal University
Graduate Institute of Rehabilitation Counseling

| Reviewers' Comments | Response from the authors |
|---|---|
| Reviewer #2: | |
| Table 3, a, b, c to be denoted in the table footnote. n for Pre, Po1, Po2 to be stated. | The a, b, c, in table 3 is denoted in the table footnotes. The n for Pre, Po1, Po2 is stated in table 3 is stated. (Line 476—477) |
| Line 430, adjustment with multiple comparisons, Bonferroni to be stated. | The Bonferroni is stated in line 421. |
| Figure 2. what is the small box beside re-training measurement? | The small box that the reviewer saw in figure might be format problem due to that the author is using the Apple Mac computer. The figure 2 has been check in a Microsoft computer and only essential information is presented. |
| Reviewer #3: | |

| | |
|---|---|
| Dear Authors,<br>Thank you for addressing my previous comments. The manuscript presents in much more accurate way the study. I have some additional comments to the revised version of the manuscript | Thank you for your review. The followings are the point-to-point response to your insightful comments. |
| Abstract: | |
| Line 54: Please re-phrase the results report on effectiveness of “both” interventions according to the study design. You actually can conclude about the first intervention effectiveness and about combination of the first and second together. | It has been revised as “ TMT and the combination of TMT and VGMT improved SSD patients’ cognition, ….”. (Line 51) |
| Introduction | |
| Line 69: “The neuropsychological system..” Please elaborate on connection between this sentence and the following one. Reference for this statement should be added. | The “neuropsychological system” was elaborate by connection of the two sentences (Line 68-70) and the reference is added in line 68. |
| Lines 74-75: “Additionally….” Please check wording of the sentence. It sounds less logical. | “The neuropsychological system” is used to replace “agent” and the sentence is revised on line 72—74 to make the sentences sound more logical. |
| Line 85: “balance” – please replace with balance | The “balanc” was a typo. It has been corrected as “balance”. (Line 83) |
| Line 109: double “and”. Please delete one of them. | The duplicate word “and” is deleted. (Line 108) |
| Lines 119 - 120 : “Impairments in cognitive functions usually occur during the first SSD episode” Please add more updated references. | More updated reference 29 and 30 (line 119) are listed in “Reference” on line709—716 to replace the original reference 28 and 29. |
| Lines 124-127: “scores on the Minimal 125 Mental Status Evaluation (MMSE) … functions” should be removed to the discussion section. | The sentence has been removed. (Line 123) |
| Lines 137-141: “. Furthermore, Hidese and… dysfunction” is less relevant for the study. Please delete from the manuscript. You can provide direct references on brain functioning alteration in schizophrenia. It is well established factor. | The sentence has been deleted. (Line 133) |

| | |
|---|---|
| Lines 177-179: “Therefore, the purpose of this study was to examine the differential effects 178 between traditional goal-directed multitasking activities and video games on cognition and 179 upper extremity and postural control in people with SSD.” The goal should be rephrased since the study design not allow you to draw conclusion on differentiation between two types of the training. | The purpose of the study is rephrased as “ the purpose of this study was to examine the effects of traditional multitask training (TMT) and combining TMT and …….”. (Line 172—174) |
| Lines 179-182: You should rephrase the sentence according with the understanding of your study design.<br>Measurements | “ We predicted that the TMT and combining both forms of multitasking training with an increased training dosage ……” is used to replace the sentence in the original manuscript. (Line 175, 176) |
| Lines 318-319: Please remove the report on the blinding group allocation since there is no groups in the study. | Thank your for the comments. The sentence has been deleted. The original second paragraph is combined with the first paragraph. (Line 183—185) |
| Results | |
| Line 466: “indicated that VGMT further improved TUG scores significantly…” Please state clear that it is “VGMT training following TMT training”. There is no group with VGMT training only or first, so you have no data to relay on concluding about the VGMT training only. | It is rephrased as the followings: “the pairwise comparison indicated that VGMT training following TMT training …” (Line 457) |
| Line 469: “especially the form of video games” The same comment as previous. Please rephrase. | It is rephrased as: ”especially the form of video games following tradition multitasks ($p < 0.05$).” (Line 460) |
| Line 476: “multitasking in the form of video game” – The same as previous. You can speak about combination of two interventions. Please rephrase you reports throughout the manuscript. | It is rephrased as: “ pairwise comparison showed that multitasking in the form of videogame following traditional multitasks ….” and “compared with TMT” is removed. (Line 468)<br><br>The whole manuscript is revised to show that the effects were not originated from VGMT only but from VGMt following TMT. For example, line 493. |
| Discussion | |

| | |
|---|---|
| Lines 498 – 504: The same sentence duplicated twice. Please remove one of them. | The duplicated sentence is removed. |
| Line 509: "Therefore, …." – please reword the sentence since it is not clear . "aht" - should be deleted. | "(traditional multitasking and video games)" in line 498 is revised as "traditional multitasking followed by video games" to avoid confusion.<br><br>The sentence "the training effects might be due to the multitasks nature of the CTT. The CTT requires a participants …..". to make it clearer. (Line 499, 502.)<br><br>The sentence " Therefore, the authors believe that the tasks in this test are multitasks fundamentally" is revised as" therefore, the authors believe that the tasks in CTT are multitask in nature." (Line 502, 503) |
| Lines 513-516: "The difficulty faced by people with SSD in multitasking was independent of the deficits in general processing speed, working memory, and attention." You should support this statement with statistic data. Please add statistical support for your statement. | The sentence is deleted since it was confusion and there is no statistical data supporting it. (Line 506) |
| Lines 515-517: "Conversely, multitasking difficulties …" the same comments as the previous one. | The sentence is revised: "Multitasking difficulties in these population were related to complex cognitive functions across multiple domains, including cognitive flexibility, inhibitory function, planning ability, prospective memory and attention shifting as measured by CTT." (Line 506—509) |
| Lines 536-537: "Hidese and colleagues [20] … impairment and decreased insular volume and white matter in people with SSD [17]. " Please delete this sentence since it has little contribution to the topic of the manuscript. | The sentence is deleted. (Line 528) |
| Please add to the limitations that you have small sample size. | The small sample size is added as one of the limitations of this study. (Line 616) |
