# Supplementary material for: Effects of multitask training on cognition and motor control in people with schizophrenia spectrum disorders
Source: PLoS One. 2022 Jun 30;17(6):e0264745. doi: 10.1371/journal.pone.0264745 (PMC9246115; doi:10.1371/journal.pone.0264745)
Supplement: Attachment — Submitted filename: PLOS ONE article review final.docx [file pone.0264745.s004.docx]

Thank you for the opportunity to read the article, “Effects of multi-task training on cognition and motor control in patients with schizophrenia spectrum disorders.” The issue is certainly important in terms of promoting the population of people with SCCD. The research is necessary and deals with very relevant areas in treating populations with cognitive and motor difficulties. It should be noted that the very fact that the participants completed the entire study – which is long and consists of two stages – and persevered to arrive is an achievement. Well done.

I have significant comments to make the article clearer.

| Abstract | Please clarify the aim of the study:  “The purpose of this study was to examine two types of multi-tasking training – traditional multi-tasking training and video game (VGMT)….  In the Abstract and the Introduction, you use the word “exergame” and in the discussion “video game.” |
| --- | --- |
| Introduction | Overall, the Introduction is written well, but I suggest changing the order of the issues:   1. The population – patients with SSD (SSD or SCCD as written in the Introduction?), functional dysfunction. 2. Cognitive dysfunction 3. Motor dysfunction 4. Multi-tasking activities and in SSD 5. Review of previous intervention in this population |
|  | Line 85: Citation and references for the WHO and for the diagnosis according to the DSM-5 are missing. |
|  | Line 98: Participation in daily multi-tasking activities …” I think there is a need to add some examples of daily activities that challenge this population and prevent them from being completely independent. |
|  | Line 134: Citation and references is missing for “one study …” |
|  | Line 139: Citation and references is missing for “leading to positive experience …” |
|  | Lines 144–151: Please add more about what did not work in Campos et al. intervention to better explain why the current intervention may be better and overtake their limitations. |
|  | Overall: Real-world activities are more ecological tasks that are close to actual activities that people do. The training in the two-intervention stage presents interesting tasks, but they present games and not real tasks that involve the participants, such as preparing a meal, self-care, or vocational tasks. See, for example, “Evaluation of a new serious game based multi-tasking assessment tool for cognition and activities of daily living: Comparison with a real cooking task” (Vallejo et al., 2017). |
| Methods | Line 166: DSM-IV needs citation and references . |
|  | Line 169: How did you check stand independently? |
|  | Line 172: MMSE needs a reference, and when and who did the MMSE? |
|  | Procedure: I suggest presenting it visually (in a figure). |
|  | Line 201: Three multi-tasking activities in the real world – I suggest to change activities to tasks. |
|  | Training program: I suggest bolding each task, such as **“The first task …”**, or putting each task in a separate paragraph. |
|  | Line 214: The third *activity* – replace the word activity with the word task. |
|  | Line 218: The ball kicking *activity* – replace with *task*. |
|  | Please clarify if the intervention was an individual or group intervention. |
|  | The rationale for selecting the tasks in Stage 1 and Stage 2 is not clear enough. Also, would it not have been worthwhile to do tasks that were parallel and more similar to each other in each of the stages? |
|  | Measure, Line 263: The full name of IADL and ADL must be written out the first time these acronyms are used. |
|  | The ABC: I suggest reporting the reliability in the current study. |
|  | Body height and body weight: What is the rationale for using those measures? How do they contribute to the study? |
|  | The BBT: I suggest reporting report the reliability they achieved in the current study (mainly because this is the first study that uses the BBT as an outcome measure with SSD)  For “The first study,” consider writing, “as well as we know [or, “as far as we could determine …”] this was the first study …” |
|  | The CTT and TUG: Psychometric properties are missing from the description. |
|  | Statistical analysis: I suggest moving the sentence that begins with “SPSS …” to the beginning of the paragraph. |
| Results | Line 374: CTT2 number error should be replaced with CTT2 Cue. It is advisable to remain consistent with the terms. |
|  | Line 389: “The result shows that all …” I suggest to write “most of” or “the majority of.” |
|  | The BSO TPL was not significant. It is not reported in the results. |
| Discussion | The subjective confidence of balance does not appear in the first sentence with the other study variables. |
|  | Lines 404–405: This is the first time you mention the two different levels. It needs to be mentioned earlier to aid understanding of this part of the Discussion. |
|  | Line 406: “significant effect of all outcome” I suggest to write “most of” or “the majority of.” |
|  | Line 417: EF should be defined in the Introduction. |
|  | Line 418: Color Trail Test = CTT. |
|  | Lines 451–452: To my understanding, the present study examined differences rather than associations, so it is not clear how this statement can be made. |
|  | Lines 477–478: “Future research is necessary to determine the effect of the multi-task full-body training model used in this study on the structure of the prefrontal area and motor cortex”. This sentence is not clear enough. In what way does it connects to the results of the present study? |
|  | Lines 488–489: Maybe use a questionnaire or other diagnostic tool that examines participants’ actual day-to-day functioning, such as the number of falls during and after training. It may be worthwhile for the activities in the training itself to be activities required of participants daily. |
|  | Lines 506–507: Is the intention to improve in the current study or in general? Because in the present study, no participation was examined. |
|  | Line 521: Which effect? |
| Figure 1 | At the stage of the analysis, there should be 27 participants because 3 were added and 3 were not yet dropped (also in the CONSORT Flow Diagram). |
