# Supplementary material for: Effects of multitask training on cognition and motor control in people with schizophrenia spectrum disorders
Source: PLoS One. 2022 Jun 30;17(6):e0264745. doi: 10.1371/journal.pone.0264745 (PMC9246115; doi:10.1371/journal.pone.0264745)
Supplement: Attachment — Submitted filename: Response to Reviews.docx [file pone.0264745.s005.docx]

The Cover Letter and Responses to Reviews

Dear Academic Editor and Reviewers:

Thank you for your review and the resubmission opportunity. The authors have revised the manuscript according to the PLOS ONE’s style requirements. The table has been included as part of the main manuscript and the individual files of the tables uploaded to the submission system initially have been removed. Finally, the ethic statement is moved and appears in the Methods section of the manuscript.

The following table shows the authors’ point to point response to each reviewer’s (Reviewer #1, Reviewer #2 and Reviewer #3) comments. Each comment is highly appreciated and the authors response to each comment with careful consideration.

Thank you very much for your review and looking forward to hearing from you soon.

Best Wishes,

Jen-Suh Chern, PhD, Associate Professor

National Taiwan Normal University

Graduate Institute of Rehabilitation Counseling

| Reviewer #1: | Authors Responses | |
| --- | --- | --- |
| Thank you for the opportunity to read the article, “Effects of multi-task training on cognition and motor control in patients with schizophrenia spectrum disorders.” The issue is certainly important in terms of promoting the population of people with SCCD. The research is necessary and deals with very relevant areas in treating populations with cognitive and motor difficulties. It should be noted that the very fact that the participants completed the entire study – which is long and consists of two stages – and persevered to arrive is an achievement. Well done. | The authors thank the reviewer #1 for the positive feedback. It is indeed important to find effective intervention program to improving the cognition and motor control in people with schizophrenia. This is the first study using multitasks as intervention program and find positive results of improving cognitive and motor control ability simultaneously. The findings are valuable for both the people with schizophrenia and clinicians. | |
| Abstract |  | |
| Please clarify the aim of the study:  “The purpose of this study was to examine two types of multi-tasking training – traditional multi-tasking training and video game (VGMT)….  In the Abstract and the Introduction, you use the word “exergame” and in the discussion “video game.” | The purpose of this study is revised in line 43—45. “ Exergames” is deleted and “video games” is used uniformly throughout the manuscript . | |
| Introduction |  | |
| Overall, the Introduction is written well, but I suggest changing the order of the issues:   1. The population – patients with SSD (SSD or SCCD as written in the Introduction?), functional dysfunction. 2. Cognitive dysfunction 3. Motor dysfunction 4. Multi-tasking activities and in SSD 5. Review of previous intervention in this population | The authors are very thankful to the Reviewer #1’s positive feedback and suggestion. However, the original order of the issues in the Introduction is preserved after considering the flow of each section. In addition, the authors would like to emphasize that the reason why multiple tasks can simultaneously improve the performer's cognitive and motor control functions is because multitasks has the nature of requiring the performer to invest in both cognitive and motor control capabilities.  SCCD was a typo and is revised into SSD. SSD is used uniformly throughout the manuscript. | |
| Line 85: Citation and references for the WHO and for the diagnosis according to the DSM-5 are missing. | The sentence is revised as “Schizophrenia spectrum disorder (SSD) is a severe mental disease and is prone to relapse, causing disability due to functional limitation.” (Line 90, 91) and a reference (Guilera G, Gomez-Benito J, Pino O, Rojo JE, Guesta MJ, Martinez-Aran A., Safont G, Tabares-Seisdedos R,. Vieta E, Bernardo M, Crespo-Facorro B., Franco M., & Rejas J. (2012). Utility of the World Health Organization disability assessment schedule II in schizophrenia. Schizophrenia Research, 138 (2-3): 240-247) is added to the reference and cited in line 91. is cited. | |
| Line 98: Participation in daily multi-tasking activities …” I think there is a need to add some examples of daily activities that challenge this population and prevent them from being completely independent. | An examples of daily multitasking activity, such as “work-related activities” is added in line109. | |
| Line 134: Citation and references is missing for “one study …” | “one study …” was a typo. Actually, the author meant “no study….”. It has been revised in line148 | |
| Line 139: Citation and references is missing for “leading to positive experience …” | A reference (Sun T-L, Lee C-H (2013) An Impact Study of the Design of Exergaming Parameters on Body Intensity from Objective and Gameplay-Based Player Experience Perspectives, Based on Balance Training Exergame. PLoS ONE 8(7): e69471. doi:10.1371/journal.pone.0069471 is cited in line 155 and added to reference list as no. 45. | |
| Lines 144–151: Please add more about what did not work in Campos et al. intervention to better explain why the current intervention may be better and overtake their limitations. | The movement required with the video game used in Campos et al.’s study is added in line 163 and the reasons for the inconclusive outcomes of this study are raised in line 174 and 175. Therefore, our study hypothesis is better supported. | |
| Overall: Real-world activities are more ecological tasks that are close to actual activities that people do. The training in the two-intervention stage presents interesting tasks, but they present games and not real tasks that involve the participants, such as preparing a meal, self-care, or vocational tasks. See, for example, “Evaluation of a new serious game based multi-tasking assessment tool for cognition and activities of daily living: Comparison with a real cooking task” (Vallejo et al., 2017). | Thank you for Your comments and reference. | |
| Methods |  | |
| Line 166: DSM-IV needs citation and references . | The reference for DSM-IV (American Psychiatric Association. Diagnostic and Statistical Manual of Mental Disorders DSM-5. 5^th^ Edition. 2013. American Psychiatric Publishing: Washington, DC, USA.) is cited in line 195 and added to the reference listed as no. 51. | |
| Line 169: How did you check stand independently? | The ability of SSD patients to stand independently was checked by therapists’ observation of participant daily performance. (Line 199) | |
| Line 172: MMSE needs a reference, and when and who did the MMSE? | A reference of MMSE was cited in line 202 and added to reference as no. 52.  The MMSE was administered to all the participants in this study by the same one physician at the facility where the study was conducted within one week before the training began. (Line 202—204) | |
| Procedure: I suggest presenting it visually (in a figure). | Figure 2 is added to visually present the study procedure. (Line 236) | |
| Line 201: Three multi-tasking activities in the real world – I suggest to change activities to tasks. | The “activities” has been changed to “tasks”, as shown in line 239. | |
| Training program: I suggest bolding each task, such as **“The first task …”**, or putting each task in a separate paragraph. | Each task was bolded as **The first task** in line 240, **The second task** in line 244, and **The third task** in line 253. | |
| Line 214: The third *activity* – replace the word activity with the word task. | The “activity” in “the third activity” has been replaced by “task”, as shown in line 253. | |
| Line 218: The ball kicking *activity* – replace with *task*. | The “activity” in “the ball kicking activity” has been deleted is shown in line 253. | |
| Please clarify if the intervention was an individual or group intervention. | The intervention of both stages was an individual intervention.“Due to limited space and equipment, all the interventions in stage 1 were performed on an individual basis” was added in line 264—265 and “The stage 2 intervention was also conducted on an individual basis” was added in line 308. | |
| The rationale for selecting the tasks in Stage 1 and Stage 2 is not clear enough. Also, would it not have been worthwhile to do tasks that were parallel and more similar to each other in each of the stages? | The following rationale of selecting the tasks in stage 1 was added in line 271—276  The three tasks selected in the first stage were goal-directed task that occupational therapist often used as intervention modalities, to improve patients’ functional performance…………. However, no formal research has reported their effects on people with SSD.  The rationale for selecting the tasks in stage 2 is added in line 309—314.  “The reason for using the two video games was that both have goal-oriented multitasking characteristics, requiring not only the participants’ exertion of cognitive resources but also appropriated movement responses of the upper and lower limb and the trunk based on the cognitive information inputs. We, therefore, believed …. motor functions.  The author choosing two different tasks in the two stages of intervention was hoping that the video game in the second stage can strengthen the motivation of participants to continue participating in training. As stated in line95—96 , people with schizophrenia are usually not motivated participating in routine intervention tasks similar to those tasks in stage 1. | |
| Measure, Line 263: The full name of IADL and ADL must be written out the first time these acronyms are used. | The full name of ADL (Activities of Daily Living) and IADL (Instrumental Activities of Daily living) were written out in line 321. | |
| The ABC: I suggest reporting the reliability in the current study. | The ABC is a self-reported, standardized instrument and its psychometric characteristics were established as cited in line 334—340. We did not examine its reliability in the current study. | |
| Body height and body weight: What is the rationale for using those measures? How do they contribute to the study? | The body height was reported to affect the amount of center of pressure shifting during quiet standing. Therefore, it was used to normalize the center of pressure related parameters (Line 413). The body weight was reported as a background data and did not contribute to this study. | |
| The BBT: I suggest reporting report the reliability they achieved in the current study (mainly because this is the first study that uses the BBT as an outcome measure with SSD)  For “The first study,” consider writing, “as well as we know [or, “as far as we could determine …”] this was the first study …” | The BBT was only tested one time at Pre, Po1 and Po2 to avoid practicing effects and to better represented the performance. Therefore, we cannot report the reliability in the current study.  The sentence began with “The first study” is revised into” As well as we know, this is the only study to ……….” , as shown in line 352. The lack of examination of the BBT reliability is reported as a research limitation in line 635 and 637. | |
| The CTT and TUG: Psychometric properties are missing from the description. | The evidence of the validity of CTT was replaced by report of psychometric characteristics of CTT in line 367—370. The evidence of the psychometric properties of that of TUG was reported in line 380—381. Appropriate references were cited. | |
| Statistical analysis: I suggest moving the sentence that begins with “SPSS …” to the beginning of the paragraph. | The “SPSS….” is moved to the beginning of the paragraph titled “Statistical analysis” as shown in line 421. | |
| Results |  | |
| Line 374: CTT2 number error should be replaced with CTT2 Cue. It is advisable to remain consistent with the terms. | The CTT2 “number error” is replaced with CTT2 “Cue” as shown in line 448. | |
| Line 389: “The result shows that all …” I suggest to write “most of” or “the majority of.” | The use of “all” is replaced with “the majority of”, as shown in line 473. | |
| The BSO TPL was not significant. It is not reported in the results. | The BSO TPL was not significant and is reported in line474. | |
| Discussion |  | |
| The subjective confidence of balance does not appear in the first sentence with the other study variables. | The subjective confidence of balance is added in the first sentence with other study variables, as shown in line 493. | |
| Lines 404–405: This is the first time you mention the two different levels. It needs to be mentioned earlier to aid understanding of this part of the Discussion. | To avoid confusion, the sentence is revised as: “Functional mobility was measured objectively by using TUG and subjectively by using ABC” in line 495—496. | |
| Line 406: “significant effect of all outcome” I suggest to write “most of” or “the majority of.” | “significant effect of all outcome” is revised as “significant effect of the majority of the outcome measures”, as shown in line 498. | |
| Line 417: EF should be defined in the Introduction. | “Executive function is a collective term for cognitive flexibility, inhibitory function, and action planning function” is added on line 97—98.  “These multifaceted cognitive functions are collectively called executive function.” is added in line 517. | |
| Line 418: Color Trail Test = CTT. | All Color Trail Test after it was first mentioned in line 356 is replaced by CTT. | |
| Lines 451–452: To my understanding, the present study examined differences rather than associations, so it is not clear how this statement can be made. | The sentence is revised as” The study results revealed that the 2 stages training combined TMT and VGMY can improve not only the cognition but also the upper limb motor function in people with SSD who have both cognition an upper limb motor deficit.” in line 546—549. | |
| Lines 477–478: “Future research is necessary to determine the effect of the multi-task full-body training model used in this study on the structure of the prefrontal area and motor cortex”. This sentence is not clear enough. In what way does it connects to the results of the present study? | “Interventions to improve the postural control and cognitive function in people with SSD have be studied separately. The present study found that training with either TMT or BGMT could simultaneously improve cognition and posture control in people with SSD, indicating that postural control is associated with cognition” is added on line 572—575 to emphasize our view and the sentence “The association between upper limb motor functional and cognition is, therefore, proved in this study.” is deleted. | |
| Lines 488–489: Maybe use a questionnaire or other diagnostic tool that examines participants’ actual day-to-day functioning, such as the number of falls during and after training. It may be worthwhile for the activities in the training itself to be activities required of participants daily. | This is a cross-sectional study and the incidence of fall is difficult to follow because no wearable device was available. Due to the finding of the study, the tasks use in this study has been incorporated into daily intervention program for the people with SSD. Future study should incorporate long term follow-up of fall incidence to answer the question. | |
| Lines 506–507: Is the intention to improve in the current study or in general? Because in the present study, no participation was examined. | The “participation” here means the participation in the training tasks during the two stages of training but not the participation in daily tasks. To avoid confusion, the “participation” here is replace by “engagement” as shown in line 610 and 611. | |
| Line 521: Which effect? | We meant the long-term effect of the tasks used in this study as intervention. The “long-term” is added in line 627. | |
| Figure 1 |  | |
| At the stage of the analysis, there should be 27 participants because 3 were added and 3 were not yet dropped (also in the CONSORT Flow Diagram). | The Figure 1 and the CONSORT chart has been corrected. | |
| Reviewer #2: |  | |
| The manuscript entitled ‘Effects of multi-task training on cognition and motor control in patients with schizophrenia spectrum disorders’ with the aim to examine the effects of traditional multi-task (MT) training and exergames as a new form of MT on cognition and motor control performance in patients with Schizophrenia spectrum disorder (SCCD). | The title of this manuscript is revised as: Effects of multitask on cognition and motor control in people with schizophrenia spectrum disorders. | |
| Methods Line 182-183, the sentence ‘The data of those who dropped out after the stage 1 training was expelled from the analysis.’ not clear. In Figure 1, it was stated n=25 were analyzed. | There are several mistakes in figure 1 and they are corrected. | |
| Line 183-184, more information for the sample size calculation to be provided i.e. outcome measures, mean/sd or %, 1 or 2-tailed test etc. | More information for the sample size calculation is provided in line 215—219. | |
| Line 276 & 280, italicized r or r to be standardized.  Statistical analysis Line 360, exact name for the type of repeated measures ANOVA to be stated. Line 361, proper citation of SPSS including publisher name to be stated. | The italicized r is standardized as *r* in line 337 and line 339.  The exact name for the repeated measure ANOVA was written as “repeated measure analysis of variance” in line 422.  The publisher of SPSS is IBM since 2009. The publisher “IBM, Chicago, USA” is added in line 421. | |
| Line 362, 0.058 ≦ ηp2 and 0.058 ≦ ηp2 < 0.138 requires revision and include 0.058 ≥ ηp2. | 0.058 ≦ ηp2 is a typo and is corrects as ηp2< 0.058 in line 432. |  |
| Type of post hoc comparison and adjustment to multiple comparison to be stated. | The default post hoc pairwise comparison was used and is stated in line 429.  The adjustment to multiple comparison is stated in 425—429. |  |
| Results Table 1, mean ± sd to be displayed first before ranges. Ranges to be denoted in the footnote as minimum to maximum value. Typo error for age figures 59.6111.46. Symbol ~ and unit Kgw to be replaced with dash (-) and kg respectively.  Table 2, N for Pre, Po1 and Po2 to be stated. What the symbol a, b, c refers to be denoted in the table footnote and in superscript form in table. Post hoc comparison of group and time to be clearly denoted. Technically, p value cannot be zero (.000) (to use symbol < and denoted with symbol ***). Decimal points for the figures to be standardized. Likewise, with Table 3.  Line 372, for CTT1T, CTT2T, T to be separated/spaced out.  Line 382, 388, 395, the figures to be cited from table.  The results could be further explored by stratifying according to age group and gender.  Some references did not comply to the journal format.  Clinical trial registration number to be stated in the manuscript once received. | The table 1 is revised according to the reviewer# 1’s suggestion in line 442.  The table 2 is revised according to the reviewer#2’s comments in line 454—455.  The T is separated from CTT1 and CTT2 as shown in line 447  The figures are cited from table 2 or table 3 in line 446, 468, 472.  The age and gender effects on the outcome measures were not the purpose of this study. Therefore, the results were not explored by stratifying according to age group and gender.  The references is revised.  The clinical trial registration number is stated in line 187. |  |
| Reviewer #3: |  |  |
| Dear Authors, The manuscript focuses on important, under investigated issue of motor functioning among people with schizophrenia. However, there are two major concerns with this study. First, there is no theoretical background for definition of the first provided intervention as multitasking. Within general classification of the interventions, the first intervention is a classic motor training, without multitasking. The intervention with the computer games somehow can fall under the definition of multitasking, but also need to be explained. Second, the study design provides a base to do some conclusion about effectiveness of motor training in general, with little ability to compare between two types of the interventions. The findings somehow support effectiveness of the traditional motor training, but did not support any conclusion about the effectiveness of videogames training. There are some additional issues, such as properness of the statistical analysis and English spelling. I strongly recommend addressing these issues first, followed by reorganization of the discussion sections in order to consider this manuscript for the publication. Some detailed comments are attached bellow. | Dear Reviewer#3:  Thank you for your comments. Based on your comments attached below, we have provided a clear and detailed definition of multiple tasks (Line 64—71). The definition states that multitasking requires participants to engage in sensory-perception and cognitive abilities as well as motor control abilities at the same time to perform goal-oriented functional behaviors. The three tasks that need to be completed in the first stage also require participants to demonstrate appropriate motor behavior according to the goal of the tasks, through processing of perception and cognitive information. Therefore, we believe that these three tasks are of multitasks in nature. The rationale of selecting the tasks in stage 1 and stage 2 training proposed in line 271—276 and 309—314, and the detailed description of the methods to complete those tasks proposed in line 240—260 and 289—303 all together construct the theoretical background for the tasks used in this study as multitasks.  Secondly, we agree with your statement that the design of this study cannot differentiate whether two different types of multitask training have different effects on the study participants, nor can it be concluded on the training benefits of video games. Therefore, we have revised the research purpose of this study. The revised research purpose is “examining the effects of combining traditional multitask training (TMT) and video games--a new form of multitask training (video game multitask training”; VGMT)--on cognition and motor control performance in people with SSD.” of people with schizophrenia (Line 43—46 and line 177—179).  Please refer to the following description for Point-to-point responses to your comments. The English spelling is edited, and the discussion is reorganized.  Thank you again for your insightful comments. |  |
| Abstract: Line 47: Please add standard deviation of the age. Line 48: TMT – you use this acromion first time. Please spell it. | Abstract  The standard deviation of the age was added in line 48.  The full spelling of TMT (traditional multitask training) is added in line 44. |  |
| Introduction The definition of multitasking is missing. Line 66: “neuropsychological and adaptive motor behavior function” The difference between the neuropsychological resources and motor adaptive resources is not clear. In order to perform motor adaptation we need neuropsychological functions. The resources may be presented using Canadian model: cognitive/neurological, emotional, motor, etc.  Line 67: Replace the word “responsibilities” with “role”. Additional editing for proper English is needed through the manuscript.  Line 71: “satisfying people's pursuit of the quality of life” should be replace with “enabling to obtain quality of life” ; “Sharpening” should be replace with “amelioration”  Lines 73-78: The phrasing makes it difficult to understand your explanation of the multitasking. Each point should be presented separately and further explained. In addition, the references should be added.  Lines 80-84: Please add example of multitasking intervention, describing it in brief.  Lines 85-86: The reference should be added  Lines 87-88: Hallucinations and delusions are not conceptualized currently as cognitive disorders. Please rephrase.  Lines 92: Please avoid usage with “patients with SSD “ and use “people with SSD” instead.  Line 94: “ The collective deficits” - not clear what you mean, cumulative?  Lines 95-96: Please add prevalence of falls in people with schizophrenia and age-specific characteristics in comparison to healthy controls.  Line 114: MMSE – should be spelled out  Line 115: MMSE less sensitive to difficulties with executive functions. Please address this issue.  Lines 134-135: The last part of the sentence is not clear. Please rephrase to support explicitly the rational of your study.  Lines 143-144: “Moreover…” Provide details on the improvement in the emotional state and add the reference.  Line 147: The name of software for training should be added.  Line 184: It is not clear how the sample size was calculated. What basic data was used to do the calculation? What sample size you find? If the calculation was done a priory or after the study completion? How it is related to your actual sample size in the study.  Line 190: “posttest: do you mean post-training?  Lines 200-226: The intervention that you provide at the first stage can be hardly considered as multitasking. You describe the standard motor training, but not multitasking. Please provide strong theoretical base for your claim that this intervention is a multitasking. The intervention did not involve simultaneously several tasks or several sensory systems. | Introduction  The definition of multitasking is added in line 64—66. (Multitasking, defined as simultaneously performing more than on task as rapidly and accurately as possible, is involved in many real-world activities. It usually occurs in an unstructured context.)  The confusion between neuropsychological resources and motor adaptive resources is revised in line 66—71. (People required nervous system resources, usually sensory-perceptual, cognitive and motor control resource, to …. which execute motor behaviors.)  The first paragraph (Line 64—89) has been revised extensively, and English has been edited by professional English editing company.  The sentence has been revised and “maintaining quality of life in the real work” is used. (Line 72—73)  “Sharpening” is replaced with “amelioration”. (Line 73)  The characteristics and definition is presented in detail and appropriated references are cited. (line 64—81) reasonable to hypothesize that the devotion of mental effort in multitasking is more than in single tasking context.  Examples of multitasking intervention are added in line 87—88. (Functional motor tasks (such as walking) with added cognitive tasks (such as counting down from 100 by 7s).  The sentence has been revised and the reference (Guilera G, Gomez-Benito J, Pino O, Rojo JE, Guesta MJ, Martinez-Aran A., Safont G, Tabares-Seisdedos R,. Vieta E, Bernardo M, Crespo-Facorro B., Franco M., & Rejas J. (2012)is added as reference no.16 (Line 91)  The sentence is rephrase as: The people with SSD initially experience thought disorganization, hallucination, and delusion, and gradually, develop deficits in both…(Line 92—93)  All the “patients with SSD “ is replaced by “people with SSD” throughout the manuscript, for example in Line 2 and 100.  Executive function is defined in line 97 as” a collective term of cognitive flexibility, inhibitory control and action planning. “ The “collective deficits” is rephrase as “these deficits” in line 99.  The prevalence of falls in people with schizophrenia and age-specific characteristics in comparison to healthy control is reported in line 104—107 and a reference no. 24 is cited in line 107.  MMSE is spelled out as: Minimal Mental Status Examination in line 125 and its abbreviation MMSE is put in a parentheses.  “However, MMSE is less sensitive to difficulties with executive functions” is added in Line 127—127.  The original sentence is rephrased in line 145—155 to better support our rationale of using the magnitude of postural sway as a measure of postural control.  The details of Compo, Mesquita, Morques, Trigueiro, and Roch’s study and its finding is further elaborated and reference no. 50 is cited. Please to line 159—161 for details.  The name of the gaming software (Grape Harvist) is added in line 164.  The method of the calculation of the sample size is stated in line 215—220. The basic data used to calculate the sample size was statistical power of 0.8, confidence level of 95%, marginal error of 0.05 with the size of population of 61 in the facility. Base on these data, the calculated sample size was 27. However, only data of 25 persons was used for statistical analysis. Therefore, the inferential statistic was adjusted as stated in the “Statistical analysis” in line 425—428.  The “posttest” is revised as “post-training evaluation.” (Line 227). The first post-training evaluation is represented by the abbreviation “Po1” (Line 227) and the second post-training evaluation is represented by the abbreviation “Po2.”(Line 230)  According to the definition of multitasking in line 64—71, multitasks are tasks that the performer demonstrates motor skill according to the simultaneous input of perceptual, sensory, and/or cognitive information. The tasks in the first stages required the participants perform motor skills (such as targeted bean bag tossing, targeted ball kicking) and at the same time processing the visual-spatial and/or visual information of the task’s setup. Therefore, the authors considered that the tasks used in the stage 1 training are multitasks in the real world.  An explanation of the reason that the authors chose the three tasks used in the study as multitasks is added in line 271—276. |  |
| Results – Please provide information on data distribution. If the distribution is different from normal, you should present the data with median and inter-quartile range, since mean and SD are not appropriate. Moreover, if the data was not distributed normally, bootstrapping should be performed before applying repeated measures ANOVA. | As stated in line 426—429. The number of participants in this study was less than the calculated sample size required. Therefore, before determine the significance of repeated measure ANOVA used in this study, the spherical hypothesis was examined with Mauchly test. When the spherical hypothesis is violated, the Greenhouses—Gaiser test which adjust the degree of freedom for calculation of the p values was used. The superscript *#* shown in table 2 and table 3 indicated the measures that violate the spherical hypothesis and the calculation of *p* was based on the adjusted degree of freedom to decrease the probability of the type I errors. |  |
| Discussion The main limitation of the study design that you had no real control for effectiveness of any intervention. In other words, the third measure (Po2) may be either a result of video-game training or a continuous improvement following traditional motor training. This point should be clearly presented in the manuscript.  You use in discussion the term video-game first time. This is good definition for the second intervention. Please replace with this term the term “exergames” that you used previously.  There is a lot of information through the discussion that should be moved to introduction section (e.g. lines 411-418). Please focus the discussion on your findings.  CTT is not multitasking test, CTT-B require cognitive shifting. Please be more specific with your writing. “CTT … as an importance outcome measure of multitasking abilities.” In addition you should rephrase your statements for proper English.  Line 442: “clinical effort devoted to upper limb motor function training in SSD has not been reported, yet” This point is not clear.  Lines 490-492: Please rephrase your conclusion since the study design did not suppotd this statement. | The reviewer is correct about the limitation of this study. Therefore, the purpose of this study is revised in line 43—46 and line 178—181. Limitation of not having the real control in this study is clearly presented in line 619—620.    The “exergames” is replaced by video game throughout the manuscript.  Thank the reviewer’s comments. The information cited in the discussion is to support the results of this research. In order to clearly present this logic, the result of this research have been moved to the first sentence of each paragraph in Discussion and followed by supporting information from previous studies. For example, the second paragraph of the discussion in line 505—533.  CTT requires the subject to use cognitive ability to judge the input perceptual information (including color and number sequence) while performing the motor skill (that is, holding a pen and tracing the trail). Therefore, the authors believe that the tasks in this test are multitasks in nature. Therefore, to complete this test quickly and correctly, the subjects need the multitasking ability.  The manuscript is edited by the professional English editing company.  What the author wants to express is that the lack of upper limb motor function has been proved to be a problem for people with SSD, and it also seriously affects the people’s performance in daily activities. However, upper limb motor function training is not the focus of clinicians, and experimental research reported the effects of training programs of any kind on improving the motor function of people with SSD patients.  To better express the authors view points, The sentence has been revised as ” no study has been reported on the clinical effort devoted to upper limb motor function training in people with SSD.” (Line540—541.) The author also added that the results support the effectiveness of combined TMT and VGMT training on not only cognition but also the upper limb motor function of people with SSD who demonstrated to have both cognitive and upper limb motor deficits.  The conclusion has been revised in line 594—596 to make the conclusion consistent with the research findings. |  |
